# Human Leukocyte Antigens -DQA1 and -DQB1 Alleles in Patients With Common Warts

**DOI:** 10.7759/cureus.18933

**Published:** 2021-10-20

**Authors:** Grazia Sánchez-Barrientos, Elisa Vega-Memije, Cristina García-Corona, Juan C Cuevas-González, Beatriz Zavaleta-Villa, Aurora Ibarra-Arce, Angelica Olivo-Diaz

**Affiliations:** 1 Department of Dermatology, Hospital General "Dr. Manuel Gea González", Mexico City, MEX; 2 Department of Dermatology, Hospital General “Dr. Manuel Gea González”, Mexico City, MEX; 3 Instituto de Ciencias Biomédicas, Universidad Autónoma de Ciudad Juárez, Mexico City, MEX; 4 Department of Molecular Biology and Histocompatibility, Hospital General "Dr. Manuel Gea González", Mexico City, MEX; 5 Department of Molecular Biology and Histocompatibility, Hospital General “Dr. Manuel Gea González”, Mexico City, MEX; 6 Department of Molecular Biology and Histocompatibility, Hospital General "Dr. Manuel Gea Gonzalez", Mexico City, MEX

**Keywords:** polymorphism, association, hpv, hla, skin warts

## Abstract

Introduction

The human papillomavirus induces the formation of lesions in different epithelia. Several studies describe an association of class II human leukocyte antigen with genital lesions, implying that they could also be related to the presence of common warts. The goal of this work was to determine the frequency of human leukocyte antigens (HLA)-DQA1 and HLA-DQB1 in Mexicans with common warts.

Methods

Thirty-two patients with a diagnosis of common warts, without any other systemic disease, and 100 healthy subjects from the same geographic area were recruited. The second exon of the HLA-DQA1 and HLA-DQB1 loci was typed by dot-blot and chemiluminescence.

Results

Alleles DQA1*03:01:01 (P = 0.021) and DQB1*03:02 (P = 0.036) were associated with the presence of skin warts. DQA1*04:01-DQB1*04:02 (P = 0.009) and DQA1*03:01:01-DQB1*03:02 (P = 0.044) were the most frequent haplotypes in patients.

Conclusion

In conclusion, the results of our study showed that the alleles DQA1 *03:01:01, DQB1*03:02, DQA1 *04:01, and DQB1*04:02 were associated with susceptibility to common warts, while DQA1*05:01 was significantly diminished in them. Consequently, the haplotypes DQA1*04:01-DQB1*04: 02 and DQA1*03:01:01-DQB1*03:02 were found to be associated with susceptibility, and DQA1*05:01-DQB1*03:01 increased significantly in controls. Therefore, the alleles of the DQA1 and DQB1 genes that are associated with susceptibility could be presenting human papillomavirus (HPV) peptides to T lymphocytes that activate a Th2-type response (anti-inflammatory cytokines), which allows the development of skin warts in this population.

## Introduction

Cutaneous warts are benign proliferations of the skin, of which common warts (*verrucae vulgaris*) and plantar warts (*verrucae plantaris*) are the most common [1]. The clinical course ranges from mild discomfort to a chronic condition that can occur in immunosuppressed adults and less frequently in immunocompetent adults. It is a common skin condition in children up to adolescence, where its incidence gradually decreases with age [2]. For example, in a study of Bacillus Calmette-Guérin (BCG) therapy in 30 patients with common non-genital skin warts, the ages at which they occurred were: five to 14 (36.67%), 15 to 24 (30%), 25 to 34 (23.3%). 35-44 (6.67%) and 45-54 years (3.3%) [3]. Up to one-third of all primary school children have warts, with two-thirds resolving spontaneously within two years [4]. Several studies on skin warts among schoolchildren around the world show a prevalence ranging from 2.4% to 33% [5]. In Mexico, in 10,000 consultations of pediatric patients (0-17 years) with skin conditions in the periods from 1971 to 1975 and from 1994 to 2003, viral warts presented 8.4% and 6.6%, respectively, without a description of their type and location in patients [6].

Warts are caused by infection with human papillomavirus (HPV). More than 170 HPV types, distributed over five genera, have been described based on their DNA sequences [7]. HPV 2, 7, 27, and 57 from the α genus, HPV 4 and 65 from the γ genus, and HPV 1 from the μ genus have most frequently been detected in cutaneous warts [8].

T lymphocytes are the main cells involved, both in cell-mediated immunity and humoral immunity. T cells recognize antigen on the surface of antigen-presenting cells (APCs) in the context of major histocompatibility complex (MHC) molecules; CD4+T cells recognize exogenous antigens presented by major histocompatibility complex (MHC) class II molecules, while CD8+ T cells recognize endogenous antigens presented by class I molecules. Activation of CD4+ T cells results in the production of Th1 cytokines or Th2, which are specific and different. A large infiltrate of T cells (both CD4+ and CD8+) and macrophages has been found on histological examination of regressing genital warts. The cytokine milieu is dominated by proinflammatory cytokines such as IL-12, tumour necrosis factor α (TNF-α), and interferon gamma (IFN-γ), which is characteristic of a Th1-based immune response, and there is an enhanced expression of CD25, intercellular adhesion molecule-1 (ICAM-1) and human leukocyte antigen DR isotype (HLA-DR) on keratinocytes, and E-selectin and vascular cell adhesion molecule 1 (VCAM1) on endothelial cells [9]. Similar findings have been described for regressing plane warts, with the presence of a mononuclear infiltrate in the lesions and healing after treatment [10].

Alleles of human MHC (human lymphocyte allele [HLA]), which are involved in the presentation of foreign antigens to immune cells, particularly the DRB1 and DQB1 class II genes, are important in the induction of the T-cell-mediated immune response against bacteria and other pathogens [11]. A number of studies have described the relationship of HPV and the HLA in genital warts, showing association with DQB1*03 alleles [12-14]. A recent meta-analysis from 22 published studies showed that HLA-DQB1*02, *03, and *06:03 had a significant association with decreased cervical cancer risk. In contrast, DQB1*05, *03:01, and *04:02 conferred a significantly higher risk to cervical cancer, supporting that HLA-DQB1 alleles may contribute to the genetic susceptibility of this disease [15].

With regard to common warts, there are few studies related to HLA, so the aim of this study was to investigate the association of the HLA-DQA1 and -DQB1 alleles in a sample of Mexican patients with nongenital skin warts caused by HPV.

## Materials and methods

Cases and controls

In this case-control study, thirty-two unrelated patients with non-genital cutaneous warts, previously diagnosed and treated in the dermatology Service, and 100 healthy, immunocompetent, unrelated subjects, without a history of having suffered from common warts, belonging to the same geographic area as patients were included. The type of HPV was determined by sequencing in an external commercial laboratory. The study complies with the current health laws of Mexico and was approved by the Ethics in Research as well as the Research Committees of the Hospital General “Dr. Manuel Gea Gonzalez.” Written informed consent was obtained from every participant.

DNA extraction and typing of HLA-DQA1 and HLA-DQB1

DNA was obtained from 10 mL EDTA-peripheral blood, using the proteinase K and phenol/chloroform extraction protocol [16]. DNA typing of the second exon of DQA1 and DQB1 genes was performed, using the polymerase chain reaction (PCR) with the primers, probes, and conditions according to the 12th International Histocompatibility Workshop [17]. The PCR amplified DNA was blotted onto nylon membranes, which were hybridized with digoxigenin-11-ddUTP-labelled sequence-specific oligonucleotide probes; after stringent washing, membranes were incubated with anti-digoxigenin antibody alkaline phosphatase labeled (Roche, Penzberg Germany); hybridization patterns were obtained by chemiluminescence detection on X-ray films [17].

Statistical analysis

Allele frequencies (AF) were calculated by direct counting, and the number of individuals positive for each allele was compared between patients and controls using Chi-square analysis with the Yate’s correction. Relative risk was calculated as an odds ratio (OR). Ninety-five percent confidence intervals (CI) were obtained by using Cornfield’s approximation. When an allele was significantly increased or diminished in the patients, etiologic (EF) and preventive (PF) fractions were calculated, respectively.

## Results

The characteristics of patients and controls are shown in Table 1. No statistical significance was found between gender and presence of warts. Since only five patients showed planar warts, we decide to group them with those of common warts, to do a general association analysis on cutaneous warts and HLA.

**Table 1:** Characteristics of patients and controls N/A: not applicable

	Female	Male	Mean age	Minimum-maximum	Common wart	Planar wart	Common and planar warts	Previous presence of skin warts
Patients	13	19	23	6-66	31	5	3	40.6%
Controls	46	54	28	3-40	N/A	N/A	N/A	0%

Five types of HPVs were detected among patients, being the most frequent HPV11 (34.4%), followed by HPV2 (25.0%) and HPV3 (21.9%). HPV26 and HPV 27 were found in a lower proportion (3.1% each), and 12.5% of the patients were negative for HPV. No association between the HPV type and HLA alleles was found (see Table 2).

**Table 2:** HVP and HLA-DQA1-DQB1 alleles of patients with common warts Chi-square Analysis:*P= 0.989; **P= 0.973; HPV: human papillomavirus; HLA: human leukocyte antigens

Allele	HPV					
DQA1*	Negative	Type 2	Type 3	Type 11	Type 26	Type 27
*01:01/4	0	1	0	3	0	0
*01:02	1	1	0	1	0	0
*02:01	0	1	0	1	0	0
*03:01:01	5	6	7	7	1	1
*04:01	1	4	5	8	1	1
*05:01	1	3	2	2	0	0
DQB1**						
*02:01	0	1	0	1	0	0
*03:01	3	3	3	1	0	0
*03:02	3	6	6	8	1	1
*04:02	1	4	5	8	1	1
*05:01	0	2	0	2	0	0
*06:01	0	0	0	1	0	0
*06:02	1	0	0	1	0	0

Allele frequencies of DQA1 and DQB1 for patients and controls are listed in Table 3. Alleles DQA1*03:01:01 (P = 0.021) and DQB1*03:02 (P = 0.036) were associated with the presence of skin warts, as a significant increase in patients was observed. Alleles DQA1*04:01 (P = 0.009) and DQB1*04:02 (P = 0.009) also showed association with susceptibility. Allele DQA1*05:01 was significantly increased in the controls (P = 0.019).

**Table 3:** Association of HLA-DQA1 and -DQB1 with common warts ^†^Allele frequency, *P-value with Yates correction, ^§^Odds ratio (95% confidence interval), ^‡^Etiologic fraction, ^#^Preventive fraction. Characters in bold and italics indicate association. EF: etiologic, PF: preventive, AF: allele frequencies, HLA: human leukocyte antigen

Allele	Patients (n = 32)			Controls (n = 100)					
DQA1	No	AF^†^ (%)		No	AF (%)	P*	OR (95% CI) ^§^	EF^‡^ (%)	PF^#^ (%)
*01:01/:04	4	6.25		21	10.50	0.444	0.62 (0.20-1.88)		
*01:02	3	4.69		20	10.00	0.290	0.50 (0.15-1.74)		
*02:01	2	3.13		14	7.00	0.407	0.51 (0.11-2.33)		
*03:01:01	27	42.19		52	26.00	0.021	2.07 (1.15-3.74)	21.8	
*04:01	20	31.25		31	15.50	0.009	2.48 (1.29-4.76)	19.6	
*05:01	8	12.50		56	28.00	0.019	0.38 (0.17-0.86)		16.7
DQB1									
*02:01	2	3.13		20	10.00	0.141	0.35 (0.08-1.55)		
*03:01	10	15.63		50	25.00	0.166	0.57 (0.27-1.21)		
*03:02	25	39.06		49	24.50	0.036	1.98 (1.09-3.59)	19.3	
*04:02	20	31.25		31	15.50	0.009	2.48 (1.29-4.76)	18.6	
*05:01	4	6.25		17	8.50	0.753	0.78 (0.25-2.41)		
*06:01	1	1.56		1	0.50	0.979	3.14 (0.19-50.96)		
*06:02	2	3.13		14	7.00	0.407	0.51 (0.11-2.33)		

Haplotype frequencies are listed in Table 4. Haplotype DQA1*03:01:01-DQB1*03:02 was the most frequent in patients, and was associated to susceptibility (P = 0.044), as well as DQA1*04:01-DQB1*04:02 (P = 0.009). Haplotype DQA1*05:01-DQB1*03:01 (P = 0.047) was of protection because it was significantly reduced in patients.

**Table 4:** Frequency of the representative DQA1-DQB1 haplotypes ^†^Haplotype frequency, *P-value with Yates correction, ^§^Odds ratio (95% confidence interval),^ ‡^Etiologic fraction, ^#^Preventive fraction. Characters in bold and italics indicate association. EF: etiologic, PF: preventive, HF: heart failure, HLA: human leukocyte antigen

Haplotype	Patients (n = 32)			Controls (n = 100)					
	No.	HF^†^ (%)		No.	HF (%)	P*	OR (95% CI)^§^	EF^‡^ (%)	PF^#^ (%)
*01:01/:04-*05:01	2	3.13		17	8.5	0.242	0.42 (0.09-1.87)		
*01:02-*06:02	2	3.13		14	6.5	0.481	0.56 (0.12-2.53)		
*02:01-*02:01	1	1.56		10	5.0	0.402	0.43 (0.05-3.41)		
*03:01:01-*03:01	3	4.69		1	0.5	0.072	7.57 (0.77-74.10)		
*03:01:01-*03:02	25	39.06		50	25.0	0.044	1.93 (1.06-3.49)	18.8	
*04:01-*04:02	20	31.25		31	15.5	0.009	2.48 (1.29-4.76)	18.6	
*05:01-*02:01	1	1.56		9	4.5	0.487	0.48 (0.06-3.83)		
*05:01-*03:01	7	10.94		47	23.5	0.047	0.42 (0.18-0.98)		13.1

## Discussion

Skin warts are a prevalent skin disease caused by various subtypes of HPV. The immunity of the host is important for its manifestation and anatomical location. Studies have implicated cell-mediated immunity as a pivotal reaction to this type of diseases, and the major histocompatibility complex class II antigens are the media for antigen presentation and development of an efficient adaptive immunity [9].

There is an enormous knowledge about HLA implications in cervical cancer [12-14,18,19]; however, little is known about nongenital skin warts and the major histocompatibility complex association. There are only three reports about the association of HLA and common warts; the first one determined the distribution of HLA-DQA1, -DQB1, and -DRB1 alleles in patients presenting HPV 2/27/57-induced common warts. The patients showed higher allele frequencies of DQA1*03:01, DQB1*03:01, DRB1*07, and DRB1*09 than controls, and lower allele frequencies of DQA1*05:01, DQB1*06:03, DRB1*01, and DRB1*03 [20]. In the second report, the Immunogenetics variation was investigated, accounting for HPV susceptibility in HIV-positive patients. The alleles HLA-B*44 and HLA-DQB1*06 and the haplotype HLA-B*44-HLA-C*05 were more frequently identified in HIV-positive patients with warts than in controls [21]. The third report is about Mexican patients in which HLA-DRB1*03 and DRB1*09 were identified for susceptibility and HLA-DRB1*06 for protection to skin warts caused by HPV infection [22]. The published study of common warts in a German population describes the HLA-DQA1*03:01 allele associated with the carriership frequency in subjects, which is in similar to our results [20]. However, in the German population, the associated DQB1 allele was *03:01, which is different in our population, since the allele associated with the disease is DQB1*03:02. These data suggest a difference in the allele frequency of the HLA-DQB1 locus in the two populations, probably due to ethnic differences.

The alleles HLA-DQA1*04:01 and HLA-DQB1*04:02 also have a significant association with the presence of warts, as well as the haplotype DQA1*04:01-DQB1*04:02 which is significantly increased in the patient group. On the other hand, the allele HLA-DQA1*05:01 was associated with protection to skin warts, with a preventive fraction of 16.7%, which is the fraction of the potential total load of the disease which is prevented by the marker of protection and/or factors associated with it.

None of the HPV detected was associated with any HLA allele or haplotype; the most frequent genotypes among our patients were HPV11 (34.4%), HPV2 (25.0%), and HPV3 (21.9%), which differs from Japanese patients with common warts, where the frequency was 44.1%, 16.4% and 14.1% for HPV1a, HPV4, and HPV65, respectively [23]. In Spaniard patients, the most prevalent genotypes detected among 105 analyzed plantar warts were HPV57 (37.14%), HPV27 (23.81%), HPV1a (20.95%), HPV2 (15.24%), and HPV65 (2.86%) [24]. In a Dutch primary school in which one-third of the children have cutaneous warts, HPV2, HPV27, and HPV57 were most frequently detected (27%, 32%, and 14%, respectively), whereas HPV1 was only found in two plantar warts, and the most prevalent types in clinically normal skin were HPV1 (59%), HPV2 (42%), HPV63 (25%) and HPV27 (21%) [25].

It is generally accepted that common cutaneous HPV types, such as HPV types 2 and 57, and less frequently types 1, 4, and 7 cause benign papillomatous cutaneous proliferations such as *verruca vulgaris*. However, in our study, a higher frequency was detected in HPV11, which is generally considered a low-risk HPV type due to its rare presence in HPV-related cancers in humans, especially cervical cancer [26]. HPV11 and HPV16 are also etiologically associated with approximately 20% of nongenital warts and 30 to 40% of laryngeal papillomas [27] and are commonly found in about 87% of patients with oral lichen planus. These two genotypes, together with HPV 6, also usually produce oral leukoplakia [28]. Nevertheless, the high prevalence of HPV11 in our patients, with respect to other populations, could be due to ethnic differences and/or some environmental factors, such as malnutrition, which could influence the infection by certain viral genotypes.

In summary, our findings suggest that HLA-DQA1 and -DQB1 polymorphisms are involved in genetic susceptibility to common skin warts in Mexican patients, and given the limited publications about common warts and HLA association, our work may contribute to the population database of genes related to this disease.

We found the following limitations to the present study: (a) given that our sample is small, it is necessary to replicate this study with a larger sample size since this limitation did not allow us to find an association between the HLA alleles and the patient's HPV types; (b) there should be conducted HLA and common warts association studies on different ethnic groups, in order to determine differences and similarities.

## Conclusions

In conclusion, the results of our study showed that the alleles DQA1 *03:01:01, DQB1*03:02, DQA1 *04:01 and DQB1*04:02 were associated with susceptibility to common warts, since were significantly increased in patients, while DQA1*05:01 was significantly diminished in them. Consequently, the haplotypes DQA1*04:01-DQB1*04: 02 and DQA1*03:01:01-DQB1*03:02 were found to be associated with susceptibility and DQA1*05:01-DQB1*03:01 increased significantly in controls. Therefore, the alleles of the DQA1 and DQB1 genes that are associated with susceptibility, could be presenting HPV peptides to T lymphocytes that activate a Th2-type response (anti-inflammatory cytokines), which allows the development of skin warts in this population.
